# The role of phase separation and related topography in the exceptional ice-nucleating ability of alkali feldspars†

**DOI:** 10.1039/c7cp04898j

**Published:** 2017-11-15

**Authors:** Thomas F. Whale, Mark A. Holden, Alexander N. Kulak, Yi-Yeoun Kim, Fiona C. Meldrum, Hugo K. Christenson, Benjamin J. Murray

**Affiliations:** a School of Earth and Environment, University of Leeds Leeds LS2 9JT UK t.f.whale@leeds.ac.uk; b School of Chemistry, University of Leeds Leeds LS2 9JT UK; c School of Physics and Astronomy, University of Leeds Leeds LS2 9JT UK

## Abstract

Our understanding of crystal nucleation is a limiting factor in many fields, not least in the atmospheric sciences. It was recently found that feldspar, a component of airborne desert dust, plays a dominant role in triggering ice formation in clouds, but the origin of this effect was unclear. By investigating the structure/property relationships of a wide range of feldspars, we demonstrate that alkali feldspars with certain microtextures, related to phase separation into Na and K-rich regions, show exceptional ice-nucleating abilities in supercooled water. We found no correlation between ice-nucleating efficiency and the crystal structures or the chemical compositions of these active feldspars, which suggests that specific topographical features associated with these microtextures are key in the activity of these feldspars. That topography likely acts to promote ice nucleation, improves our understanding of ice formation in clouds, and may also enable the design and manufacture of bespoke nucleating materials for uses such as cloud seeding and cryopreservation.

## Abstract 2

Alkali feldspars which are phase separated into K- and Na-rich regions nucleate ice far better than those without phase separation.

## Graphical abstract

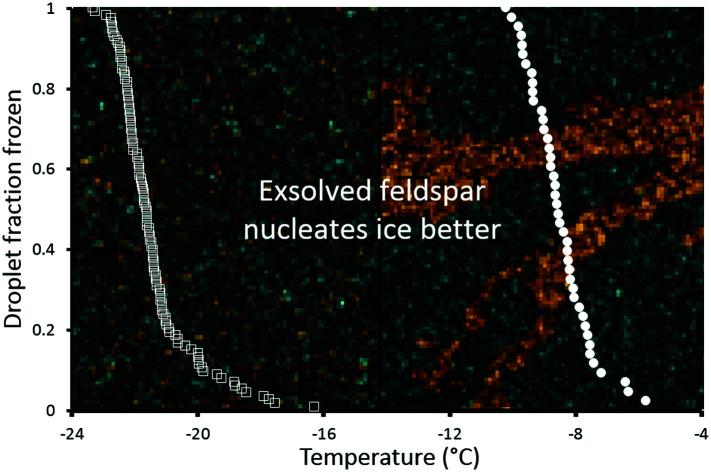

## Introduction

The formation of ice crystals in the Earth's atmosphere plays a key role in the formation and evolution of clouds, the hydrological cycle and climate.^1–4^ In the atmosphere, liquid water droplets can supercool to temperatures below −35 °C ^5,6^ but many clouds commonly contain a mixture of both supercooled water droplets and ice crystals (mixed-phase clouds) or are completely glaciated at much higher temperatures. The nucleation and subsequent growth of ice crystals in a cloud dramatically alters its radiative properties, leads to precipitation and shortens its lifetime. Much effort has therefore been devoted to quantifying the ice nucleating efficiency of the many species of atmospheric aerosol that may be responsible for glaciation of clouds. However, we do not understand why specific particle types nucleate ice more effectively than others.^7,8^

It is well-known that mineral dusts are important atmospheric ice-nucleating particles (INPs).^1,9,10^ Huge quantities (1000s Tg year^−1^) of mineral dust that are generated in arid regions such as the African and Asian dust belts are dispersed throughout the Earth's atmosphere.^11^ It has recently been established that certain alkali feldspars (which contain Na^+^ and K^+^) are the most efficient ice nucleating minerals of those common in the atmospheric dust, both in immersion mode^12–18^ and deposition mode.^19,20^ This runs counter to previous ideas, which had assigned this role to clay minerals.^1,2,12^

Alkali feldspars make up a large proportion (up to ∼30%)^12,21^ of airborne desert dust and are globally important for atmospheric ice nucleation in mixed-phase clouds.^22^ However, despite their common chemical compositions and crystal structures, the ability of feldspars to nucleate ice varies widely.^13,14^

The ability of particulates to nucleate ice has traditionally been attributed to lattice-matching between a crystal face and ice, where this may occur on planar faces or within specific sites such as cracks, pores or defects.^1,23^ Very recently it has been suggested that the ability of alkali feldspars to nucleate ice is related to the presence of high-energy crystallographic faces at steps, cracks or cavities.^24^ However, given the similarity in their structures, the same high energy faces may be expected to be exposed at the surface of both alkali feldspars and plagioclase feldspars (which contain a mixture of Ca and Na). This suggests there is another factor that also governs the ability of alkali feldspars to nucleate ice effectively.

This article describes an investigation into the ice nucleating properties of a wide range of alkali feldspar samples, where our goal was to determine the origin of their ability to nucleate ice effectively. An important property of alkali feldspars is that many have undergone phase separation (exsolve) into K-rich and Na-rich regions separated by grain boundaries. This results in microtexture known as “perthitic texture”, where this can lead to a range of surface features associated with the strain between K- and Na-rich regions. These surface features occur on a range of length scales from nanometres to millimetres^25^ and originate from chemical attack on dislocations caused by the strain between grain boundaries. We have here investigated the relationship between perthitic textures and ice-nucleating activity and demonstrated that this is key to the superior ice nucleating properties of alkali feldspars. This proves strong support for the role of specific topographical features as active sites (the spatial locations where ice-nucleation takes place) for ice nucleation.

## Experimental

**Fig. 1:**
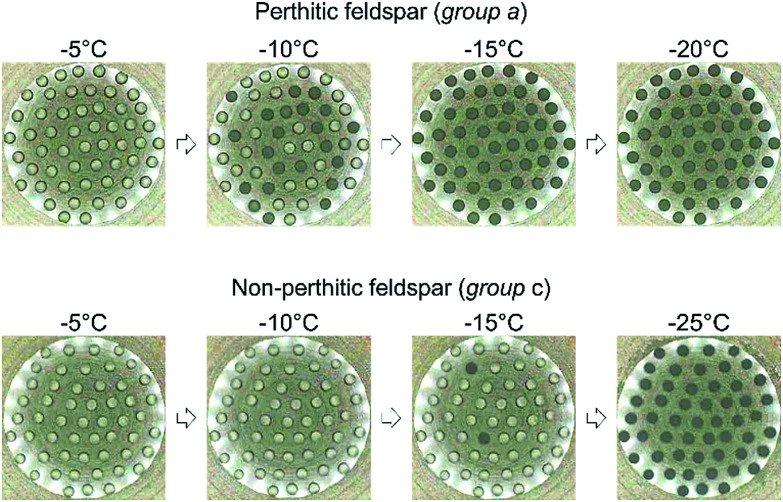
Images showing the progress of two droplet freezing experiments. Droplets contained a perthitic feldspar (top row) freeze at much higher temperatures than droplets containing the same amount of a non-perthitic feldspar (bottom row). Using these images fraction frozen curves of the type shown in Fig. 3(a) can be constructed. Since the surface area of material in each droplet is known, *n*_s_(*T*) can be determined (Fig. 3(b)). For scale, the glass slide in the image is 22 mm in diameter and each droplet is approximately 1 mm in diameter.

The μl-nucleation by immersed particles instrument (μl-NIPI) droplet freezing assay was used for all ice-nucleation experiments conducted in this study. It has been thoroughly described previously.^26^ Briefly, approximately 40, 1 μl droplets of MilliQ water (18.2 MΩ cm) containing the ice nucleator under investigation are pipetted onto a hydrophobic silanised glass slide (Hampton Research) using an electronic pipette (Picus Biohit). The slide is supported by an Asymptote EF600 Stirling Cryocooler, which is used to control the temperature of the droplets. The slide and droplets are covered by a Perspex shield and a flow of dry nitrogen over the droplets is used to prevent water condensation. Condensation is a potential problem because ice can propagate from a frozen droplet to an unfrozen droplet by the freezing of water condensed on the surface, artificially enhancing the freezing rate, however use of a flow of dry nitrogen prevents this from happening without introducing other difficulties.^26^ Freezing is monitored using a digital camera and in this study droplets were always cooled at 1 °C min^−1^. This process allows the fraction of droplets frozen at a given temperature to be determined. The progress of a typical experiment is shown in Fig. 1. Suspensions of the feldspars tested were made up gravimetrically and stirred using a magnetic stirrer bar for a few minutes prior to use. In this study we used 1 wt% and 0.1 wt% suspensions. It can be seen in Fig. 3(a) that all data presented here are well clear of the background freezing of the instrument (droplet freezing that occurs in the absence of a deliberately introduced heterogeneous nucleator). The surface areas of the samples were measured by Brunauer–Emmett–Teller (BET) nitrogen gas adsorption using a Micromeritics TriStar 3000.

The ice-nucleating efficiency of the feldspars is quantified as the number of ice nucleating sites that become active per surface area on cooling from 0 °C to temperature *T*. *n*_s_(*T*) can be calculated using:^27^1
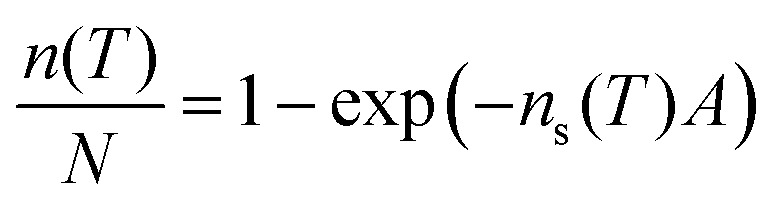
where *n*(*T*) is the number of droplets frozen at temperature (*T*), *N* is the total number of droplets in the experiment and *A* is the surface area of nucleant per droplet. *n*_s_(*T*) is a singular, site specific measure of ice nucleation efficiency which does not account for the effects of time dependence. It has been shown that ice nucleation by several feldspars is weakly time-dependent.^28^ It is important to note that in the experimental fraction of droplets frozen (*n*(*T*)/*N*) cannot be directly applied to clouds since this is an experiment specific quantity. In order to be sensitive to rare sites which are important for clouds, we tend to work with much higher particle concentrations per droplet than would typically be present in the atmosphere. The derived *n*_s_, is in principle a surface area independent quantity which can then be applied to the atmosphere given a surface area of dust in the atmosphere.^12,22^

Error bars in the derived *n*_s_(*T*) were calculated using Monte Carlo simulations of possible site distributions propagated with the uncertainty in surface area of nucleator per droplet, as described in Harrison *et al.*^14^ The temperature uncertainty for μl-NIPI has been estimated to be ±0.4 °C.^26^

Samples characterised by Hodson *et al.*^29^ were obtained from the authors of that study. All the samples, except Eifel sanidine, were the smallest particles separated from powders by the size selection procedures used by Hodson *et al.*^29^ Dry sieving was used to remove the larger particles leaving only particles smaller the 63 μm. The larger particles were used in that study, leaving the smaller material for our study. It should be noted that the Shap feldspars come from phenocrysts removed from a larger matrix so the purity of these samples may not be as high as that of the other samples. Other samples were obtained either as ground powders or as small rocks which were thoroughly cleaned then ground using an agate mortar and pestle. Samples from Hodson *et al.*^29^ used in this study were received in sealed vessels so it seems unlikely that significant contamination has occurred since grinding. Similarly, the vast majority of the surface area of the powders produced from rocks specifically for this study will have originated from the interior of the rock sample so contamination is unlikely. Finally, as part of BET analysis, samples were all heated to 110 °C. This will have removed all moisture and volatiles from the samples prior to ice nucleation experiments. See Table S1 (ESI†) for details of the feldspars used in this study.

## Results

### The importance of perthitic texture in alkali feldspars

Fifteen different alkali feldspars with a wide range of perthitic textures were selected for study. For simplicity, we have classified these samples into three broad groups: (a) samples with coarse perthitic texture (perthites); (b) samples with perthitic texture too small to resolve in an optical microscope (cryptoperthites); and (c), samples lacking perthitic texture. We have also included data for a sanidine for which we do not have microtextural information and a plagioclase feldspar that exhibits intergrowth structures. Full details of all samples and descriptions of feldspar structure and perthitic texture are given in Table S1 and Supplementary Notes 1 and 2 (ESI†).

**Fig. 2:**
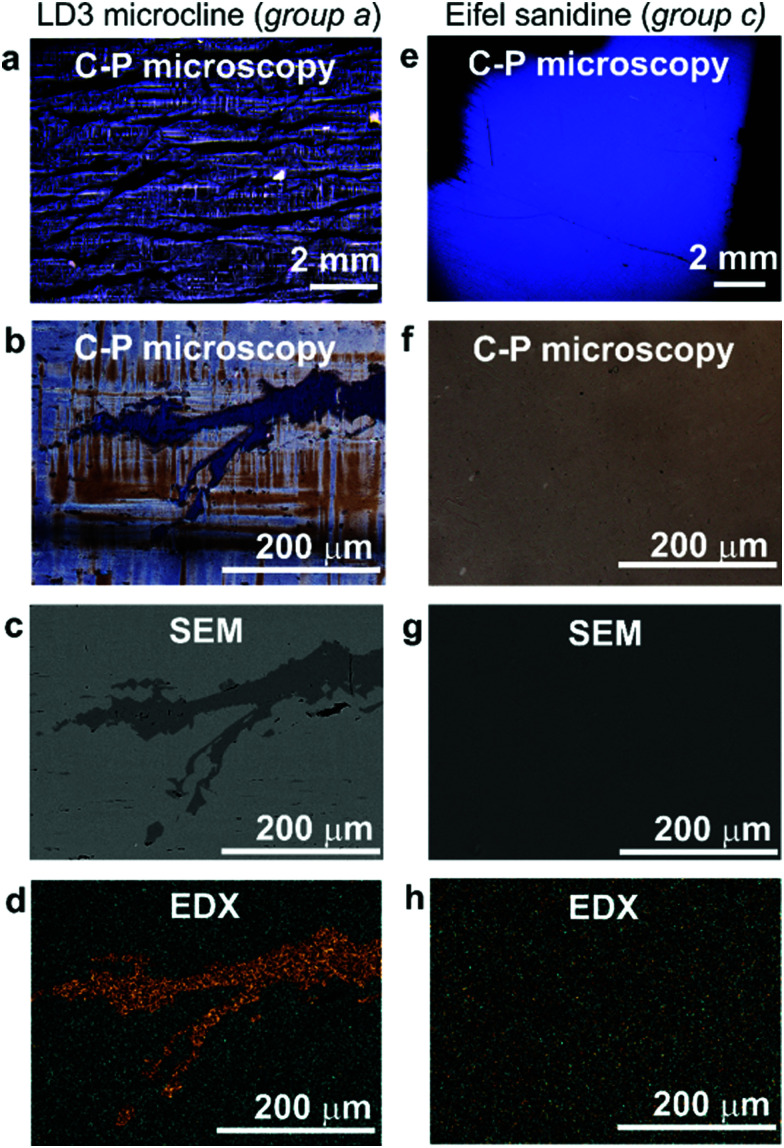
Images illustrating the striking difference between samples with and without perthitic structures. The two samples have similar compositions and similar crystal structures, but, the LD3 microcline (a–d) sample has undergone a phase separation into Na- and K-rich regions, whereas the Eifel sanidine (e–h) sample has not. These thin sections are orientated on the (001) plane. (a) is an (C-P) optical micrograph between crossed polarisers of LD3 microcline and (b) is a higher magnification C-P optical micrograph. Both images were taken using filters to enhance the contrast between the microcline region and the albite veins. (c) is an SEM micrograph of the same region as shown in (b), and (d) is an EDX map of the same region. In (d) orange indicates the presence of sodium and blue indicates potassium. Other elements are omitted for clarity. (e–h) are equivalent images to (a–d) respectively for Eifel sanidine. It can be seen that Eifel sanidine is non-perthitic whereas LD3 microcline has a coarse perthitic texture. In LD3 microcline the underlying tartan microtexture (the criss-cross patterning in the potassium rich regions) seen in (a) and (b) indicates that the bulk polymorph is microcline. The tartan structure originates from twinning that is a consequence of the increased Si–Al ordering that occurs during transition from monoclinic orthoclase or sanidine to triclinic microcline (see Fig. S1, ESI†). This agrees with powder X-ray diffraction analysis from Harrison *et al.*^14^ The tartan microtexture is cut through by albite veins.

While the 15 alkali feldspars tested have very similar crystal structures and compositions, their perthitic structures are very different. This is illustrated in Fig. 2, which contrasts images of a typical group a alkali feldspar (panels a to d) with images of a group c alkali feldspar (panels e–h). The group a feldspar (left hand column of images) has undergone phase separation into K and Na rich regions and exhibits perthitic texture. This is evident in the cross-polarised light microscopy (a and b) and the elemental mapping images (d). In contrast, the group c feldspar (right hand column) completely lacks these features. An image of a group b feldspar is given in the ESI† Fig. S2 in which transmission electron microscopy is used to show the presence of sub-micron perthitic features. Hence, the key physical difference between these feldspar samples lies in their perthitic structure. More detail on the nature and origin of perthitic structures in alkali feldspars is given in the Supplementary Note 2 (ESI†).

**Fig. 3:**
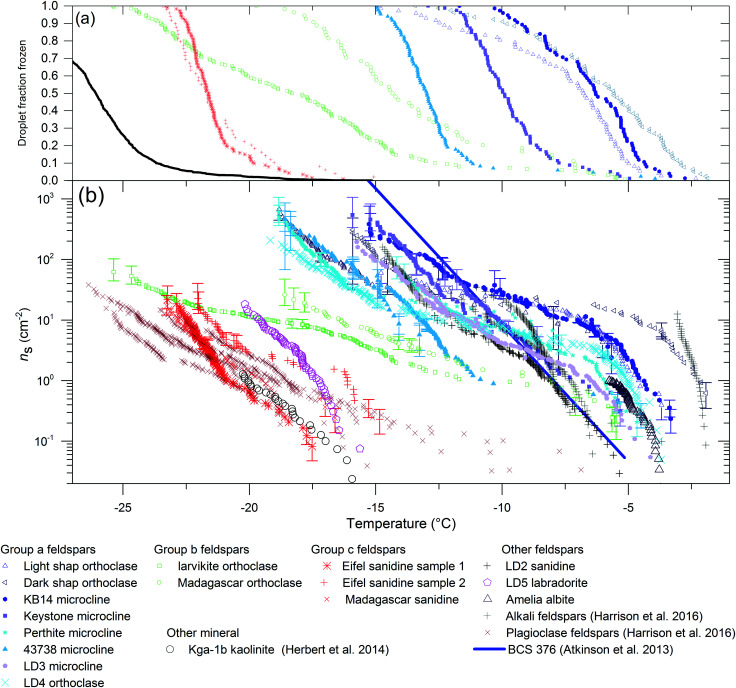
Plots showing the difference in ice-nucleating activity of feldspars with perthitic structures (group a) and those without (group c) as well as feldspars with sub-micron perthitic structures (group b). Panel (a) shows droplet fraction frozen for 1 wt% suspensions. Panel (a) also includes a line showing the temperature at which water droplets freeze on the instrument in the absence of heterogeneous nucleators. Panel (b) shows *n*_s_(*T*) for both 1 wt% and 0.1 wt% suspensions of the feldspars plotted independently (experimental conditions are given in Table S1, ESI†). *n*_s_(*T*) is the cumulative number of ice nucleating sites per unit area that have become active as a function of temperature on cooling from 0 °C to temperature *T*. The paramaterisation for BCS 376 microcline from Atkinson *et al.*^12^ is included as is data for kaolinite from Herbert *et al.*^28^ for comparison. Most of the data for alkali and plagioclase feldspars from Harrison *et al.*^14^ are plotted as faded crosses as we do not have microtextural details for these feldspars. Panel (a) also shows a fit to the background freezing for the droplet-freezing instrument used in this study. All the feldspar samples tested here nucleated ice more efficiently than the background freezing of the instrument. The *n*_s_ curve for each mineral is made up of data from multiple (between 2 and 4) droplet freezing experiments. The method of calculating error bars described in Harrison *et al.*^14^ and takes into account uncertainties associated with small numbers of freezing events.

Standard methodologies were employed to quantify the ice-nucleating efficiency of these samples. The results of the freezing experiments in Fig. 3 show that the three alkali feldspars tested that lack perthitic textures (group c, red colours) nucleate ice far less efficiently than the alkali feldspars that possess clear perthitic textures (group a, blue colours). At a given temperature, the ice-active site density on the alkali feldspar samples lacking perthitic texture (group c) is more than two orders of magnitude lower than for the group a perthitic feldspars. The two samples with very small-scale perthitic structures (green points, group b; the cryptoperthites) nucleate ice with efficiencies intermediate between the group a and group c feldspars. Some droplets freeze at warmer temperatures similar to group a feldspars, while some droplets freeze at colder temperatures. These results strongly suggest that perthitic texture is a key element in ice nucleation by feldspars.

Data on other alkali and plagioclase feldspars from Harrison *et al.*^14^ are also included in Fig. 3(b), although no information was available on the microtexture of these samples. As can be seen, plagioclase feldspars (light red points) nucleate ice with similar efficiencies to alkali feldspars lacking perthitic structure. Plagioclase feldspars generally form solid solutions and mostly lack perthitic texture (see Supplementary Note 2 (ESI†) for more details). However, while they do not generally exsolve there are several solubility gaps in their phase diagrams. These can lead to very fine (submicron) exsolution lamellae that are similar in scale to those of the group b alkali feldspars. A sample of labradorite (LD5 labradorite) that exhibits this behaviour was investigated, and although it nucleated ice slightly more efficiently than the other plagioclase feldspars tested by Harrison *et al.*^14^ it was not as efficient as the group a and group b feldspars (Fig. 3). This provides strong evidence for the link between perthitic textures and ice-nucleating properties.

Previous work has suggested that ice-nucleating ability depends on the polymorph of the alkali feldspar.^16,18^ Microcline, orthoclase and sanidine differ in the level of ordering of the aluminosilicate framework, and it was reported that the more ordered microcline can nucleate ice more efficiently than the disordered forms.^16,18^ If this were true, it would imply that slight differences in the crystal structure of feldspars could substantially influence ice nucleation efficiency. Contrary to this argument, Harrison *et al.*^14^ demonstrated that a disordered alkali feldspar (LD2 sanidine) nucleated ice as well as most ordered feldspars, this data is included in Fig. 3(b). We also show that other relatively disordered alkali feldspars (Light Shap orthoclase, Dark Shap orthoclase and LD4 orthoclase) nucleate ice highly efficiently. We therefore conclude that the ordering of the aluminosilicate framework in alkali feldspars does not directly determine their ice nucleating efficiency. To confirm the polymorphic identity of the feldspars we measured the Raman spectra of the feldspars which were identified as being highly disordered. The spectra are presented in Fig. S2 (ESI†). Raman spectroscopy provides a convenient way of assessing the bulk level of ordering in the samples as the spot size of the Raman laser is several micrometers across and so larger than the scale of exsolution microtexture in the feldspars examined here. Reference spectra are available for comparison.^30^ The level of aluminosilicate framework ordering can be determined from the shape of peak at around 460 cm^−1^, it is clear that Eifel sanidine, LD2 sanidine, Madagascar orthoclase and Madagascar sanidine are all disordered. For comparison we also measured the Raman spectrum of BCS376 microcline, which does indeed appear to be highly ordered as expected.

### Why does perthitic texture influence ice nucleation?

**Fig. 4:**
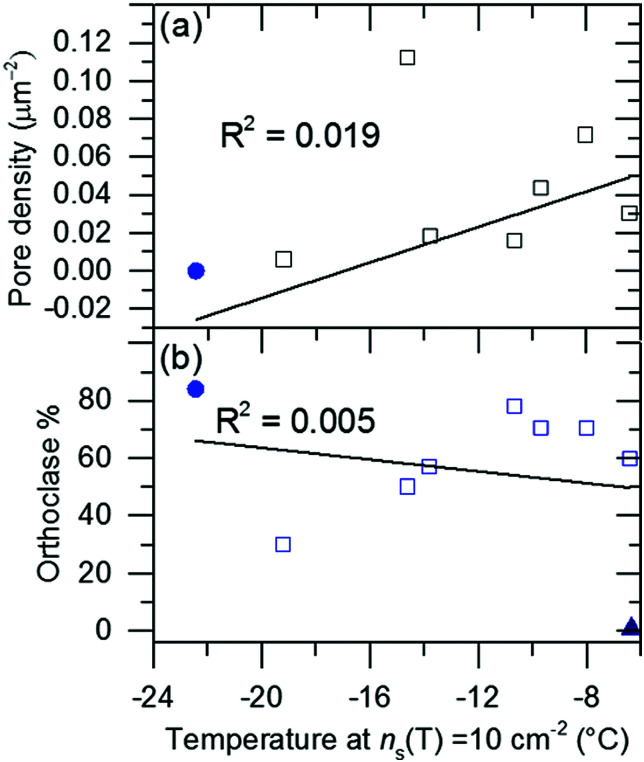
Correlation plots between feldspar properties and ice nucleating ability. Ice nucleation efficiency is approximated by the *T* at which *n*_s_ = 10 cm^−2^, with higher temperatures indicating greater ice nucleating ability. The feldspar properties, (a) micropore density and (b) orthoclase percentage. Micropore density is taken from Hodson *et al.*^29^ and orthoclase% is detailed in Table S1 (ESI†). Amelia albite, for which ice nucleation measurements were made in Harrison *et al.*,^14^ is also included in panel (b) as a triangular symbol. Filled blue circles on both plots indicate Eifel sanidine.

Fig. 3 shows that there is significant variability in efficiency of the perthitic group a feldspars to promote ice nucleation (high efficiency means a higher nucleation temperature). This provided an opportunity to correlate further known properties of these feldspars with their ice-nucleating efficiencies. Fig. 4 shows plots of the temperature for which *n*_s_(*T*) = 10 cm^−2^ (as a simple, single-number proxy for ice-nucleation activity), against micropore density and potassium percentage (K-feldspar%) for a range of perthitic feldspars, where Hodson *et al.*^29^ measured the properties for some of these samples (supplied by Prof. Mark Hodson, University of York; see Table S1, ESI†). The data show that the crystal structures and chemical compositions of efficient and inefficient ice nucleating feldspars are similar, which eliminates this as the major source of differences in activity, while perthitic texture is common to all active feldspars.

#### Dislocations

Perthitic texture typically gives rise to strain between K^+^ and Na^+^ rich domains. This results in the formation of crystallographic dislocations that can serve as sites for chemical attack by water and acids in feldspars and other minerals.^31,32^ The dislocation density varies with the domain size of the K^+^ and Na^+^ rich regions such that group a feldspars have the highest dislocation density, group b an intermediate density and group c the lowest.^25,29^ Although the group a feldspars are the most active ice nucleators, it is noted that ice nucleation sites are far rarer than dislocations. In this study, we observed ice-active site densities of ≈0.1–100 sites per cm^2^, whereas even Eifel sanidine has ≈10^6^ dislocations per cm^2^.^33^ Higher dislocation densities would be anticipated in many of the other feldspars tested. This suggests that if they are active, only a tiny fraction of dislocations create a site with the correct structure to nucleate ice.

#### Twinning

Many feldspar minerals exhibit crystallographic twinning and all microcline samples are likely to be twinned to some extent. The potential role of twinning was explored by examining the behaviour of LD4 orthoclase, where this material is not twinned on a scale observable by light microscopy (see Fig. S3, ESI†). Yet it nucleates ice well. In contrast, the LD5 labradorite sample tested here is a poor ice nucleator, despite having extensive twinning (see Fig. S4, ESI†). The alkali feldspars with well-ordered aluminosilicate frameworks (the sanidines and orthoclases) are also likely to be less extensively twinned, yet nucleate ice efficiently. Features associated with twinning therefore do not appear to promote efficient ice nucleation.

#### Micropore density

Microporosity is a characteristic property of many alkali feldspars and results from water-feldspar interactions (see Supplementary Note 2, ESI†)^25,29,34^ that typically leave a network of micropores.^35^ These have been defined by Hodson *et al.*^29^ as structures with cross-sectional areas greater than 1 × 10^−2^ μm^2^. No correlation was found between the density of micropores on the feldspar surfaces and ice-nucleation efficiency, as can be seen in Fig. 4.

#### Orthoclase percentage

‘Zolles *et al.*^17^ suggested that the cations which leach from the surface of feldspar (Ca^2+^, Na^+^ and K^+^) interact with water in different ways and can therefore influence nucleation. They suggest that Na^+^ and Ca^2+^ reduce the efficiency of nucleation whereas the presence of K^+^ has a neutral or positive influence on nucleation. Nevertheless, our work shows that features associated with microtexture are critical for nucleation, but it is possible that nucleation at these sites may be influenced by the nature of dissolved cations as suggested by Zolles *et al.*^17^ A correlation with feldspar ion content is inconsistent with our observation that there is no significant correlation between potassium content and ice-nucleation efficiency (Fig. 4). The sample of Amelia albite shown in this plot is particularly interesting, because despite containing very little potassium, it nucleates ice extremely well. However, Amelia albite does contain a low density of K-rich exsolution lamella (see ESI†), which supports our hypothesis that effective ice nucleation is related to perthitic texture. We have not included Amelia albite in the group a feldspars because of its strikingly different ice-nucleating properties. In particular, Amelia albite's ability to nucleate ice decreases strongly when it is left suspended in water, unlike the standard group a feldspars that we have examined.^14^ Overall, Fig. 4(b) indicates that there is no obvious correlation of ice-nucleating ability with K (orthoclase) content.

#### Nanoscale topographical features

Perthitic feldspars display a range of nanoscale topographical features including etch pits^36^ that are formed by chemical attack at dislocations, nano-tunnels, which appear during solution–feldspar reactions at the sites of dislocations;^37^ and ‘pull aparts’, which are nanoscale cracks found crossing albite lamellae.^37^ Many of these may also be associated with larger-scale features such as micropores. Given the absence of any link between the chemical composition or structure of the alkali feldspars and their nucleating ability, we suggest that nanoscale topographical features associated with perthitic textures provide the sites for efficient immersion-mode ice nucleation. This is very much what is to be expected – only nanoscale features of a pore or surface can possibly influence the nucleation stage. This is because the size of a critical ice nucleus (the minimum cluster size of water molecules required for the spontaneous growth of ice) is of the order of several nanometers for the degree of supercooling in these experiments. Indeed, recent work has shown that nanoscale defects in a silica surface can significantly increase the ice-nucleation frequency^38^ while micron-scale surface scratches and defects on glass, silicon and mica do not substantially affect the ice-nucleation efficiency.^38–40^ These studies indicate that it is unlikely that topographical features on length scales greatly exceeding the size of a critical nucleus could influence nucleation events.

## Discussion

The results presented in this paper show that alkali feldspars with high ice nucleating efficiency have perthitic texture, whereas feldspars that have very similar chemical compositions and crystal structures but which lack perthitic texture exhibit much lower ice nucleating efficiency. This strongly indicates that the surface topography of the feldspars is key to their high activities. The powerful influence of topography on crystal nucleation is well established in a range of systems. Deposition-mode experiments with mica surfaces have shown that nucleation of organic compounds from vapour is greatly enhanced on surfaces roughened with diamond powder.^41^ It has also been shown that crystal nucleation from vapour can occur *via* freezing of liquid condensates without the need for any supersaturation of the vapour phase.^42^ In particular, organic crystals nucleate in supercooled liquid, condensed from vapour in very acute (wedge angle ≈ 0.3°) “pockets” occasionally found at cleavage steps on mica surfaces.^43,44^ Similarly, in an atmosphere of water vapour, ice crystals were observed to form in such pockets at −39 °C,^43^ a temperature where homogeneous nucleation of ice in supercooled water is expected.^6^ There is, however, a free energy barrier associated with a crystal emerging from a pore,^43,45^ such that deposition-mode nucleation in a pore will not necessarily lead to observable ice crystals. These observations have confirmed what Fukuta suggested in 1966;^46^ that ice may deposit from vapour by freezing of supercooled water condensates, also known as “pore condensation and freezing” (PCF).^40^

Environmental scanning electron microscopy has also been used to give direct insight into deposition-mode ice nucleation on kaolinite particles.^47^ Ice crystals were found to deposit on the rough edges of the kaolinite particles below −27 °C, and the authors suggest that this was a chemical effect due to the different structure of the edges compared to the smoother basal plane. In their study of deposition-mode ice nucleation by feldspar, Kiselev *et al.*^24^ suggested that rare patches of the (100) plane, exposed by fracture or weathering in or close to surface topographies, were responsible for the observed formation and oriented growth of ice crystals, a conclusion that was supported by atomistic simulations. If this is the case, the frequency of occurrence of exposed (100) planes must vary widely between different feldspars to account for their vastly different ice-nucleating efficiency, where this could derive from differences in the way the feldspars fracture, or by variations in leaching or ageing processes.

Considering further the potential role of surface chemistry, it is well established that close lattice matching can significantly enhance ice-nucleation efficiency, as is thought to be the case for AgI.^48^ In general, the importance of lattice-matching is dependent on many additional factors and a good lattice match does not guarantee good nucleating ability.^49^ Many of the best nucleating agents have an abundance of surface hydroxyls rather than a particularly good lattice match to ice.^50^ Furthermore, on many surfaces with high nucleating ability, ice crystals depositing from vapour are often observed to form in the vicinity of cracks or surface defects.^48,50^ The precise nucleation sites are more difficult to locate in immersion-mode nucleation, but it has been suggested that surface pits formed through partial dissolution, *e.g.* at lattice defects, can increase nucleation efficiency.^49^

Deposition-mode nucleation *via* freezing of supercooled liquid (PCF) obviously has no analogue in the case of immersion-mode nucleation. However, surface roughness is still known to promote nucleation of crystals from solution^51,52^ and quantitative studies have shown that pores of the right size can greatly increase the rate of crystal nucleation from solution of small organic molecules^53^ as well as proteins.^54,55^ It is has been suggested that pores of the appropriate size (typically 1–10 nm) act to stabilise or promote the formation of critical nuclei. Such a mechanism was first discussed for the freezing of gallium by Turnbull in 1950,^56^ who suggested that certain pore sizes could prolong transient association of atoms, possibly augmented by favourable pore-atom interactions. In particular, materials with a wide pore-size distribution are the optimal protein nucleants as it increases the likelihood of encountering a pore of optimum size for a particular protein.^55,57^ We suggest that this mechanism may also operate in immersion-mode ice nucleation, where a complex interplay of topography, charge, polarity and chemical effects would determine the nucleation efficiency of each site. This would also account for the wide distribution of site efficiency, with the most active sites requiring a statistically unlikely combination of properties.

## Conclusions

In conclusion, we have shown that the exceptional ice nucleating ability of alkali feldspars is related to a phase separation into Na- and K-rich regions within feldspar crystals, known as perthitic microtexture. The strain related to the boundary between the two phases is well-known to give rise to a host of topographic features. We suggest that it is the details of this topography that can act to cause wide variations in the ice-nucleation efficiency of feldspars with very similar chemical compositions and crystal structures. We suggest that nanoscale features associated with the perthitic texture of certain alkali feldspars provide the most active nucleating sites. Since the highly active sites are very rare, it is likely that a combination of topography and surface chemistry is required. However, feldspars that lack suitable topographical features are no more effective at nucleating ice than layered aluminosilicates such as kaolinite (see Fig. 3). A better understanding of why feldspars are effective at nucleating ice may help us to identify other natural nucleating materials, as well as to design bespoke nucleating materials. This would benefit the many areas of science and technology, such as pharmaceutical production,^58^ biomineralisation,^59^ nanoparticle synthesis^60^ and cryopreservation.^61^ In the atmospheric sciences an improved understanding of ice nucleation is needed to better quantify the action of natural and anthropogenic ice-nucleating particles, as well as to design artificial ice-nucleating particles for the geoengineering of clouds to reduce global warming,^62^ or for precipitation control.^23^

## Conflicts of interest

There are no conflicts to declare.

## Supplementary Material

CP-019-C7CP04898J-s001
