# Supplementary material for: The role of phase separation and related topography in the exceptional ice-nucleating ability of alkali feldspars
Source: Phys Chem Chem Phys. 2017 Nov 15;19(46):31186–93. doi: 10.1039/c7cp04898j (PMC11970471; doi:10.1039/c7cp04898j)
Supplement: CP-019-C7CP04898J-s001 [file CP-019-C7CP04898J-s001.pdf]

Electronic Supplementary Material (ESI) for Physical Chemistry Chemical Physics.
This journal is © the Owner Societies 2017

Supplementary information for "The role of topography in the exceptional ice-nucleating ability of alkali feldspars"

Thomas F. Whale[a], Mark A. Holden[a,b], Alexander N. Kulak[b], Yi-Yeoun Kim[b], Fiona C. Meldrum[b], Hugo K. Christenson[c], Benjamin J. Murray[a]

[a] *School of Earth and Environment, University of Leeds, Leeds, LS2 9JT, UK*
[b] *School of Chemistry, University of Leeds, Leeds, LS2 9JT, UK*
[c] *School of Physics and Astronomy, University of Leeds, Leeds, LS2 9JT, UK*

This supplementary information contains details of feldspar structure, the nature and origin of perthitic structures and details of the samples used in this study.

Supplementary Note 1. Details of Feldspar structure

The structure of feldspar is discussed in detail by many texts book, for instance, Deer and Howie[1]. Here we give a brief overview. Feldspars are minerals that have the composition MT_4O_8 where T stands for atoms which are capable of tetrahedral coordination to oxygen (Al, Si) and M is a metal cation. The tetrahedra share corners in a 3-D continuous framework with the M cations filling the cavities in this structure. There are three common end-member chemical compositions, namely $KAlSi_3O_8$, $NaAlSi_3O_8$, and $CaAl_2Si_2O_8$. The top panel of Supplementary Fig. 1 shows a ternary phase diagram for the feldspar system. Members of the K-Na series are known as alkali feldspars, while members of the Na-Ca series are known as plagioclase feldspars. Alkali feldspars can adopt subtly different crystal structures (named microcline, orthoclase and sanidine) depending upon the rate at which they cool during formation and the environment they experience after crystallisation. In sanidine the Al is fully disordered, with Al and Si atoms occupying random locations in the aluminosilicate framework. Al has some intermediate ordering in orthoclase and is fully ordered in microcline, always occupying the same location in the aluminosilicate framework. This subtle difference in Al positioning means that microcline has triclinic symmetry, while orthoclase and sanidine have monoclinic symmetry, but otherwise the crystal structures are very similar. The differences between the polymorphs are not distinct, with the extent of possible ordering forming a continuum from total order of the aluminosilicate framework to total disorder. As such, the naming of minerals is somewhat subjective, particularly when differentiating

between orthoclase and sanidine. The crystal structure of the plagioclase feldspars is similar to that of the alkali feldspars, with identical arrangement of atoms[2].

Of late, there has been some debate about the role of role that polymorphism plays in the ice nucleating efficiency of feldspars[3-6]. Since the distinction between alkali feldspar polymorphs in X-ray diffraction using Rietveld refinement is subtle, we have used Raman spectroscopy to verify polymorph identification through comparison with standard spectra (see supplementary Fig. 2). It can be seen that the alkali feldspars polymorphs with more ordered aluminosilicate frameworks have more complex splitting of peaks, notably in the vicinity of 460 cm^{-1} and 280 cm^{-1}. As mentioned above, the level of ordering of the aluminosilicate framework can lie at any point on a continuum from complete order where the Al cation always occupies the same tetrahedral site (microcline) to complete disorder, where sites are randomly occupied (Sanidine). LD2 sanidine appears to have very little ordering of the aluminosilicate framework while Madagascar sanidine, Madagascar orthoclase and Eifel sanidine are all intermediate between the literature spectra for orthoclase and sanidine, with notable shoulders on the peak at 460 cm^{-1}.

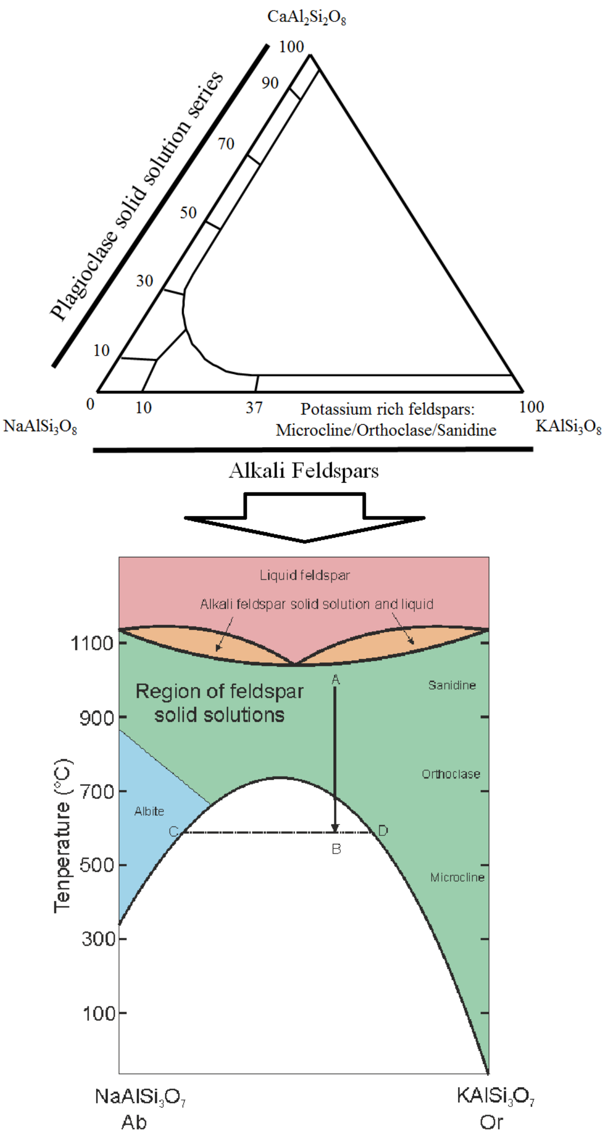

Supplementary Figure 1. Phase diagrams for Alkali feldspar. Top Panel: Ternary phase diagram for the feldspar system, adapted from Harrison *et al.*[3]. Bottom panel: Simplified, approximate phase diagram for alkali feldspars adapted from Parsons [7]. Mineral names indicate regions where the various phases of feldspar are stable. Sanidine, orthoclase and microcline are K-feldspar polymorphs that differ in the level of ordering of their aluminosilicate frameworks [2]. Albites are Na-feldspars. Below around 700°C, feldspar containing mixtures of Na^+ and K^+ decompose from a single solid solution phase into mixture of feldspar phases. The arrows indicate possible pathways. When a feldspar of composition A is cooled through the green region of the diagram any crystallization will result in a single feldspar phase. On further cooling to point B the solid solution is no longer stable. Instead, the feldspar will tend to exsolve to produce a feldspar richer in Na^+ at point C and a feldspar richer in K^+ at point D. The resulting rock will be perthitic in nature. The spatial scale of this structure will depend primarily on the temperature at which exsolution occurs and the timescale on which exsolution happens[8]. After a rock has formed further perthitic texture can be formed due to hydrothermal processes (see SI).

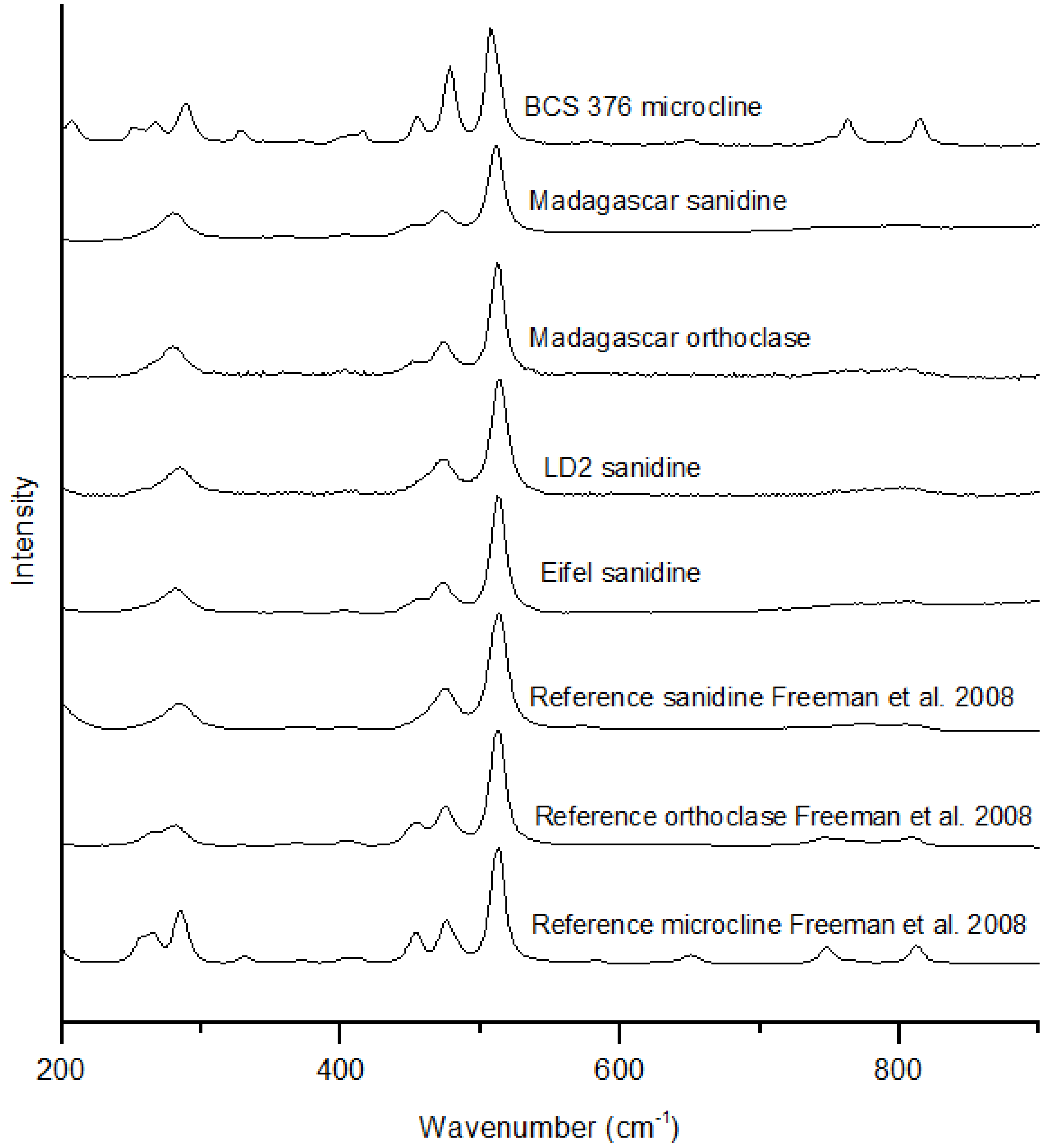

Supplementary Figure 2. Raman spectra of various feldspars investigated in this study. Reference spectra are from Freeman *et al.*[9]. A spectrum for BCS 376 microcline, used by Atkinson *et al.*[10] is also included. Spectra were collected using a Renshaw inVia Raman microscope equipped with a 514 nm laser.

Supplementary Note 2. Feldspar microtexture

Feldspars contain various features on scales from centimetres to a few nanometres, collectively termed microtexture[1]. One type of microtexture, perthitic microtexture, is common in alkali feldspars, but absent in plagioclase feldspars. Perthitic microtexture arises from the fact that solid solutions of alkali feldspars are metastable at the colder temperatures relevant to their formation in nature (see the bottom panel of Supplementary Fig. 1). The result of this is that the majority of alkali feldspars in nature are exsolved to varying extents, i.e. they are 'perthites'. This perthitic microtexture often takes the form of lamellae which are enriched in either Na^+ or K^+ ions. In nature perthitic microtexture can also be caused by deuteric and hydrothermal processes which involve dissolution of the feldspars in liquid water present in the formation environment and subsequent reprecipitation of a perthitic feldspar[8, 11]. The scale of perthitic microtexture can range from a few nanometres to centimetres, and microtextures on multiple length scales and of multiple origins are often found in a single feldspar crystal [12].

A typical example of a perthitic sample contrasted against a sample which is not perthitic is presented in Fig. 1 of the main text. Fig. 1 shows light microscope (a and b), scanning electron microscope (SEM) (c) and elemental mapping images (d) of a typical perthitic alkali feldspar. These images clearly show microtextural features where the bulk microcline (K-rich) is cut through with bands of albite (Na-rich). The microcline can be identified by its cross-hatched (tartan) twinning, which does not occur in sanidine or orthoclase as it is a product of the ordering of its aluminosilicate framework[2]. In contrast, Figures 2 e-h show similar images for an alkali feldspar, Eifel sanidine, that cooled sufficiently quickly during its formation to avoid exsolution, and which is therefore pristine (i.e. lacks any perthite microtexture). The images clearly lack the features associated with exsolution or deuteric/hydrothermal alteration and the elemental mapping image shows that the Na and K are mixed throughout the structure in a manner consistent with a solid solution.

Identification of very fine perthitic texture of the *group b* nano-perthites is not possible using microscopy of the type employed in Fig. 1. Instead to identify the nano-perthitic structure of Madagascar orthoclase we have employed transmission electron microscopy (TEM). Supplementary Fig. 2 shows these micrographs.

Microtextural information for LD2 was not available as the sample was obtained in a ground form. However, microcline is more likely to be exsolved and microtexturally complex than sanidines as the formation conditions that promote ordering of the alumino-silicate framework often promote exsolution.

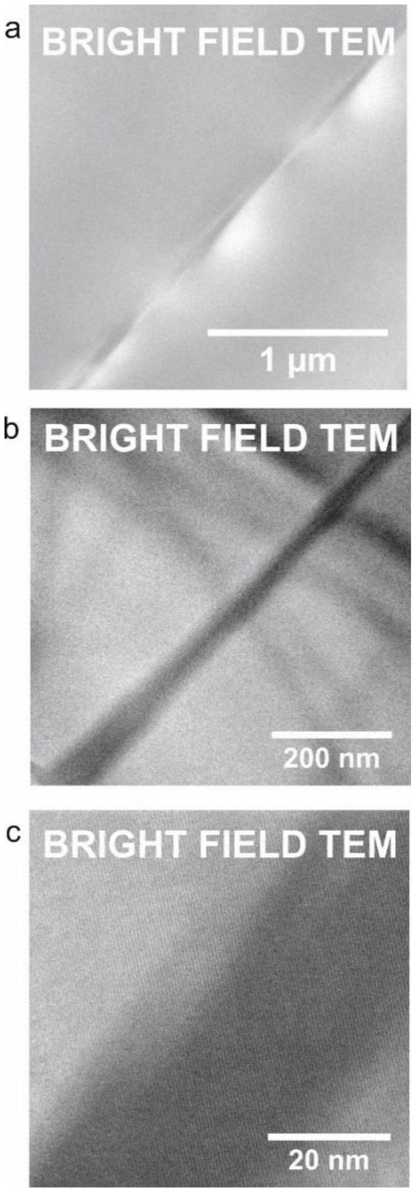

Supplementary Figure 3. Transmission electron microscope images of Madagascar orthoclase showing a fine exsolution lamella. Cross sections of the sample were prepared using Focussed Ion Beam Milling (FIB), using an FEI dual beam system, equipped with a 30 kV Ga-beam and a field emission electron gun operated at 5 kV. Ga-ion currents from 7 nA to 100 pA were applied to remove part of the crystal. A final polishing step was carried out at 30 kV and 30 pA. The samples were then characterised with bright field TEM using a FEI Tecnai TF20 FEG-TEM operating at 200 kV.

Sample name	Bulk chemical composition	Main K-feldspar mineral phases	Pristine microtexture	Pristine grain boundary type	Alteration microtextures	BET surface area (m^2g^{-1})	BET spherical particle diameter (μm)	Source
**Light Shap**	$Or_{71}Ab_{28}An_{<1}$[a]	orthoclase	lamellar microperthite	semi-coherent	yes	0.70 ± 0.01	3.2	Hodson et al.[13]
**Larvikite**	$Or_{30}Ab_{58}An_{11.5}$[a]	orthoclase	lamellar crypto-mesoperthite	coherent	no	0.77 ± 0.02	2.9	Hodson et al.[13]
**Dark Shap**	$Or_{71}Ab_{28}An_{<1}$[a]	orthoclase	lamellar microperthite	semi-coherent	yes	0.84 ± 0.01	2.6	Hodson et al.[13]
**Perth perthite**	$Or_{57.4}Ab_{42}An_{0.6}$[a]	microcline	vein macroperthite	semi-coherent	yes	0.70 ± 0.01	3.2	Hodson et al.[13]
**Keystone microcline**	$Or_{78.3}Ab_{20.7}An_{1.0}$[a]	microcline	vein macroperthite	semi-coherent	yes	0.77 ± 0.01	2.9	Hodson et al.[13]
**43738**	$Or_{50.3}Ab_{44.0}An_{5.6}$[a]	low albite/ microcline	braid microperthite	coherent	yes	0.84 ± 0.03	2.6	Hodson et al.[13]
**KB14**	≈ $Or_{40}Ab_{60}$[a]	low albite/ microcline	braid microperthite	coherent	yes	1.09 ± 0.01	2.0	Hodson et al.[13]
**LD2 sanidine**	$Or_{86}Ab_{14}$[b]	sanidine	unavailable	unavailable	unavailable	3.78 ± 0.03	0.6	Harrison et al.[3]
**LD3 microcline**	$Or_{74}Ab_{25}An_{<2}$[b]	microcline	vein macroperthite	semi-coherent	yes	1.78 ± 0.01	1.2	University of Leeds Mineral Collection
**LD4 orthoclase**	unknown	orthoclase	lamellar microperthite	Semi-coherent	no	2.34 ± 0.02	0.9	University of Leeds Mineral Collection
**LD5 labradorite**	unknown	plagioclase	Finely exsolved plagioclase	coherent	no	2.40 ± 0.035	0.9	University of Leeds mineral collection
**Madagascar orthoclase**	$Or_{90}Ab_8An_{<2}$[b]	orthoclase	lamellar crypto-perthite	coherent	yes	1.38 ± 0.01	1.6	Excalibur Minerals
**Eifel sanidine sample 1**	$Or_{84.1}Ab_{15.9}An_{0.1}$[a]	sanidine	none	none	no	2.05 ± 0.02	1.1	eBay seller rocksale247
**Eifel sanidine sample 2**	$Or_{84.1}Ab_{15.9}An_{0.1}$[a]	sanidine	none	none	no	1.89 ± 0.02	1.2	eBay seller rocksale247
**Madagascar sanidine**	$Or_{95}Ab_3An_{<2}$[b]	sanidine	none	none	no	1.54 ± 0.01	1.4	Excalibur Minerals
**Amelia albite**	$Or_1Ab_{98}An_1$[c]	albite	unavailable	unavailable	unavailable	0.80 ± 0.02	3.0	Excalibur minerals

[a]Composition of sample determined by Hodson et al.[13] using an electron microprobe. [b]Composition of sample determined by Rietveld refinement of powder XRD pattern. [c]Composition of sample determined by Carpenter et al.[14]

Supplementary Table 1. Alkali feldspars samples used in this study. Or, Ab and An are abbreviations for orthoclase, albite and anorthite respectively, which are potassium, sodium and calcium feldspar endmembers respectively. The ratios indicate the molar proportions of these components.

Supplementary Note 3: Twinning in feldspars

Feldspars often exhibit crystallographic twins. Fig. S3 shows that LD5 lacks twins while figure S4. Shows that LD4 labradorite possess twinned areas.

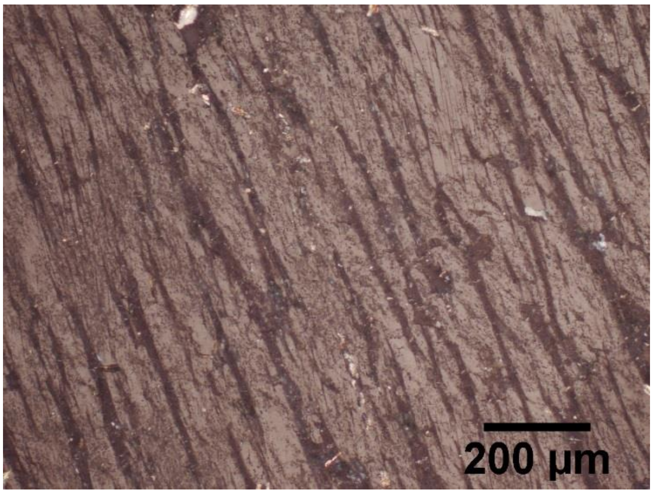

Supplementary Figure 4: Light micrograph of the (001) face of LD4 orthoclase. It can be seen that the feldpar is exsolved but lacks twinning.

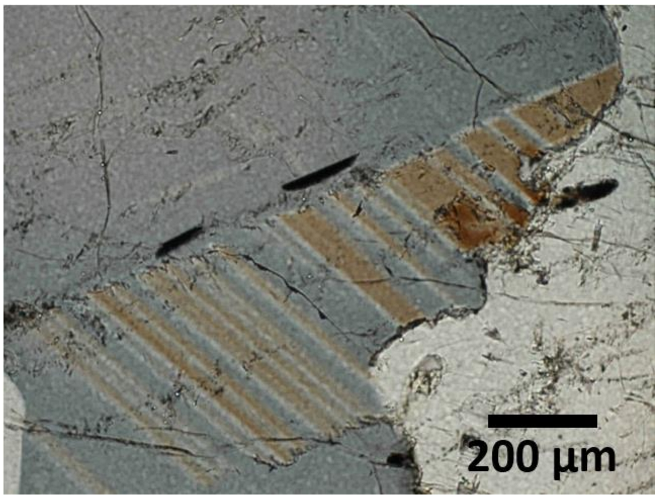

Supplementary Figure 5: Light micrograph of LD5 labradorite. Parts of the labradorite sample contain substantial twinning as can be seen in this image.

Supplementary Note 4: Amelia albite

As discussed in in the main text Amelia albite is a somewhat unusual case. It contains very little potassium and is often treated as a pristine albite. For this study we have obtained a new sample of Amelia albite from Excalibur minerals.

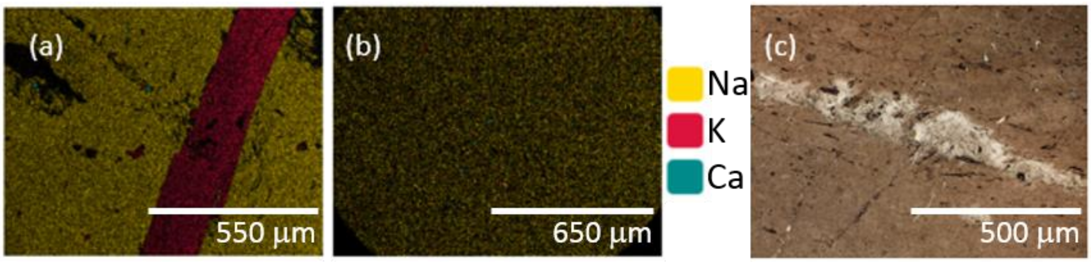

Supplementary Figure 6: EDX maps and light microscope image of amelia albite thin section. (a) EDX mapping of Amelia albite. There is some amount of phase separation of the feldspar, with discrete K rich regions running through Na rich albite. (b) EDX mapping of a more typical region of the same Amelia albite thin section. This region contains Na, with little Ca or K. (c) Light micrograph of a thin section of Amelia albite showing an exsolution lamella.

Supplementary References

1. W. A. Deer, R. A. Howie and J. Zussman, *An introduction to the rock forming minerals*, Addison Wesley Longman, Harlow, UK, 1992.
2. H.-R. Wenk and A. Bulakh, *Minerals: their constitution and origin*, Cambridge University Press2004.
3. A. D. Harrison, T. F. Whale, M. A. Carpenter, M. A. Holden, L. Neve, D. O'Sullivan, J. Vergara Temprado and B. J. Murray, *Atmos. Chem. Phys.*, 2016, **16**, 10927-10940.
4. A. Peckhaus, A. Kiselev, T. Hiron, M. Ebert and T. Leisner, *Atmos. Chem. Phys.*, 2016, **16**, 11477-11496.
5. S. Augustin-Bauditz, H. Wex, S. Kanter, M. Ebert, D. Niedermeier, F. Stolz, A. Prager and F. Stratmann, *Geophys. Res. Lett.*, 2014, **41**, 7375-7382.
6. L. Kaufmann, C. Marcolli, J. Hofer, V. Pinti, C. R. Hoyle and T. Peter, *Atmos. Chem. Phys.*, 2016, **16**, 11177-11206.
7. I. Parsons, *Mineralogical Magazine*, 2010, **74**, 529-551.
8. I. Parsons, J. D. Fitz Gerald and M. R. Lee, *American Mineralogist*, 2015, **100**, 1277-1303.
9. J. J. Freeman, A. Wang, K. E. Kuebler, B. L. Jolliff and L. A. Haskin, *The Canadian Mineralogist*, 2008, **46**, 1477-1500.
10. J. D. Atkinson, B. J. Murray, M. T. Woodhouse, T. F. Whale, K. J. Baustian, K. S. Carslaw, S. Dobbie, D. O'Sullivan and T. L. Malkin, *Nature*, 2013, **498**, 355-358.
11. M. R. Lee, K. Waldron and I. Parsons, *Mineralogical Magazine*, 1995, **59**, 63-78.
12. I. Parsons, J. Fitz Gerald, M. Heizler, L. Heizler, T. Ivanic and M. Lee, *Contrib Mineral Petrol*, 2013, **165**, 931-960.
13. M. E. Hodson, M. R. Lee and I. Parsons, *Geochim. Cosmochim. Acta*, 1997, **61**, 3885-3896.
14. M. A. Carpenter, J. D. C. McConnell and A. Navrotsky, *Geochim. Cosmochim. Acta*, 1985, **49**, 947-966.
